# Long‐lasting mood deterioration following transcranial direct current stimulation treatment for fibromyalgia: A case report

**DOI:** 10.1002/ccr3.7712

**Published:** 2023-08-10

**Authors:** Sarvenaz Rahimibarghani, Hamid R. Fateh

**Affiliations:** ^1^ Physical Medicine and Rehabilitation Department Tehran University of Medical Science Tehran Iran; ^2^ Neuromuscular Research Center Tehran University of Medical Sciences Tehran Iran

**Keywords:** fibromyalgia, long‐term adverse effects, mood changes, transcranial direct current stimulation (tDCS)

## Abstract

**Key Clinical Message:**

While tDCS has been studied as a safe and effective tool for managing pain in fibromyalgia, there is a possibility of triggered long‐lasting mood changes. TDCS may potentially negatively affect mood in specific individuals with fibromyalgia.

**Abstract:**

Transcranial direct current stimulation (tDCS) is a noninvasive neuromodulator that showed promising results in pain reduction among individuals with fibromyalgia (FM). Despite the potential benefits, it may have some adverse events that are mainly transient. However, long‐lasting effects can also occur. We presented a 31‐year‐old man whose symptoms and signs were consistent with fibromyalgia, and he received tDCS over C3 to reduce diffuse pain. Although, immediately after fulfilling the session, he became restless, agitated, and aggressive, and his symptoms lasted approximately 2 months later.

## 1 INTRODUCTION

Fibromyalgia (FM) is a common chronic pain condition characterized by widespread pain and other symptoms such as fatigue, headache, sleep difficulties, cognitive problems, anxiety, and depression.^1^

Depression and FM are two disorders that often co‐occur, and patients with FM have a high chance of suffering from major depression. There are similarities between the two disorders, such as the psychological stressors that can trigger episodes of either condition. Psychotherapy and antidepressant are used to manage both conditions, suggesting a shared pathophysiological pathway.^2^

There are various pharmacological and nonpharmacological therapeutic options that can help manage symptoms in FM. Noninvasive brain stimulation (NIBS) method such as transcranial direct current stimulation (tDCS) is proposed as an adjunct therapy to manage some symptoms in FM. TDCS involves applying a low‐intensity electrical current to the brain, which is thought to modulate the activity of cortical neurons and is considered a safe device in healthy populations; however, using it among patients with neuropsychiatric disorders may be associated with more adverse events and patients with more severe and complex conditions may be at a higher risk.^3^, ^4^ Two mechanisms of action are explained for tDCS, including immediate and long‐term effects. The instant results of tDCS are caused by changes in the electrical activity of neurons in the targeted brain region. In addition to the immediate effects, there is growing evidence to support the long‐term effects due to the changes in the structure and connectivity of the brain.^5^

Several studies showed the efficacy of left primary motor cortex (M1) stimulation in decreasing pain in FM. Effects were also long‐lasting, with the participants reporting continued pain relief for up to 4 weeks after the end of the treatment.^6^ Apart from the motor control role of M1, there are also different contributions in pain modifying and behavioral changes reported related to this area. M1 has some interactions and connections with other regions like the pain processing network, and activation of this part resulted in focal and distant neural alteration; moreover, M1 stimulation may help to modulate the perception of pain in the brain by altering the way that pain signals are processed.^7^, ^8^

Herein, we described a 31‐year‐old man with mood worsening following the application of M1‐tDCS for reducing pain in fibromyalgia, and his symptoms sustained for 8 weeks.

## 2 CASE PRESENTATION

A 31‐year‐old man came to the physical medicine and rehabilitation clinic complaining of a generalized muscle ache that started several years ago. He stated that the pain sometimes affected his sleep, and he felt unrefreshed throughout the day. The patient also reported occasional headaches and fatigue. Apart from nonsteroidal anti‐inflammatory drugs, he has not been on other medications.

He was an overweight single man and reported weight gain due to inactivity. He was a nonsmoker and did not use illicit drugs or consume alcohol. The patient had a depressed mood but did not have a significant medical or psychiatric history interfering with his daily functioning or requiring hospitalization; however, his mother suffered from a major depressive disorder and had been on antidepressants for several years.

Upon physical examination, apart from some trapezius muscle trigger points, others were unremarkable, and laboratory tests were within normal limits. The widespread pain index (WPI) was 10, and the symptom severity scale (SSS) was five. Therefore, the diagnosis of fibromyalgia was made according to the 2016 revised criteria.^9^

The patient had several appointments that were prescribed duloxetine and pregabalin, underwent acupuncture for some trigger points, and was recommended to do regular exercise. However, he has not been compliant with the previous regimen and believed those approaches were not helpful, so he was seeking another effective treatment. Alternative treatment options have been discussed, and as a result, tDCS was chosen. He was also referred to a psychiatrist for cognitive behavioral therapy or diagnosis of any accompanying psychiatric condition.

**FIGURE 1:**
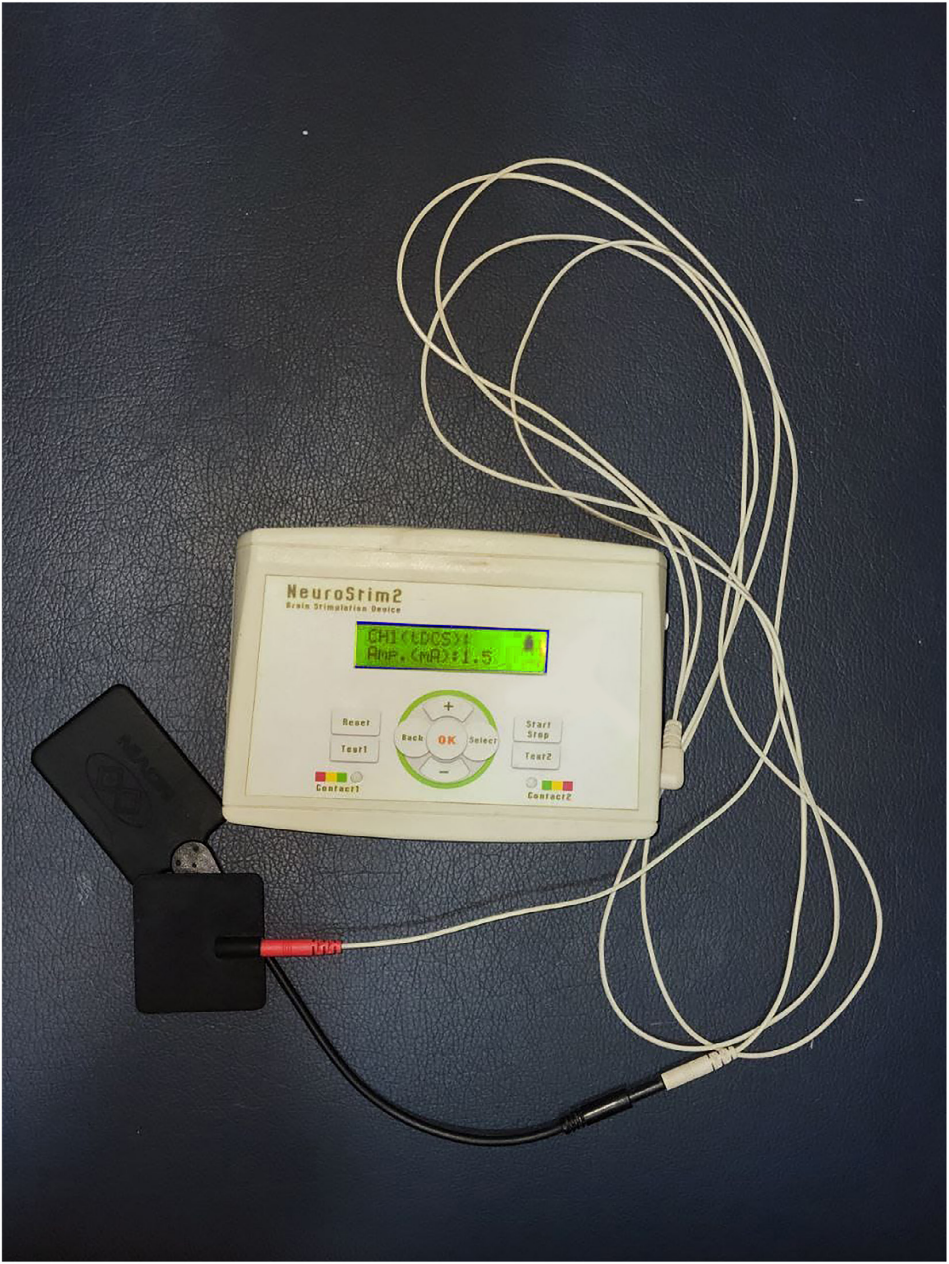
Transcranial direct current stimulation device.

In the first session, the patient received electrical stimulation via two sponge surface electrodes (4 × 4 cm [active] and 4 × 9 cm [reference]) through a 2‐channel tDCS device (Neurostim‐2, Medina Teb) (Figure 1). The anode electrode was placed over the left primary motor cortex (C3) based on the International 10–20 system, and the cathode was located on the contralateral supraorbital region (Fp2). The session lasted 20 min with an intensity of 1.5 mA. Right after the session was over, we observed that the patient was slightly irritable and agitated. Additionally, he had some headaches. We informed him that these symptoms were temporary and requested him to remain at the clinic until they subsided. Symptoms improved 1 hour later, and he left the clinic in good condition.

Review sessions were scheduled, but he did not return for visits or respond to the calls. After 2 months, his mother visited our clinic for her knee pain and informed us of behavioral changes in her son since receiving tDCS. To better understand, we requested that he return for a follow‐up visit. He arrived with his sister, who noted worsening mood, increased anxiety, verbal aggression, poor sleep, and decreased concentration were affecting her daily activities, and the family members had never seen him behave in such a manner before. However, he did not have hallucinations, delusions, or suicidal thoughts. He also had no changes in her fibromyalgia pain. Since the beginning of his symptoms, he did not seek any medical help and has not attended the psychological consultation session. He told his family that the doctor had assured him that his symptoms were temporary and would disappear on their own, but he was afraid of coming to the clinic to have a further intervention that could pose him with a newer problem.

He stated that his symptoms got better but occasionally became anxious and aggressive. A psychiatric appointment was scheduled for further evaluation of his mood symptoms. He received cognitive behavioral therapy and was started on sertraline and nortriptyline with a diagnosis of depressive disorder accompanied by fibromyalgia. Meanwhile, we contacted him regularly to detect adherence issues. He also has been swimming twice a week.

The patient was reassessed at 6 weeks follow‐up, his mood and sleep improved, and the severity of his pain decreased.

## 3 DISCUSSION

The relationship between depression and FM is complex, with some theories suggesting that depression predisposes a patient to FM, while others believe that FM triggers depression. Additionally, the hypothesis suggests that some people may be predisposed to depression and pain through a similar pathogenic pathway. Diagnosing depression in FM patients can be challenging because depression is one of the common symptoms of fibromyalgia and may be part of the diagnostic criteria; however, it can also be a separate condition that requires a particular diagnosis. In both FM and depression, central sensitization (CS) can lead to a cycle of pain and negative emotions that can exacerbate the condition.^2^, ^10^, ^11^

CS is defined as an amplification of neural signaling within the central nervous system and is a process in which the central nervous system becomes hyperexcitable and hypersensitive to sensory stimuli. This phenomenon is a crucial factor in the evolution of chronic pain in FM. Several studies have shown that patients with FM have variations in the neural processing of pain, including increased activation of pain‐related brain areas and altered functional connectivity within these regions.^12^, ^13^, ^14^, ^15^

TDCS has been shown to modulate the activity of pain‐related brain regions, including the primary somatosensory cortex, anterior cingulate cortex, and insula. The modulation of these regions results in changes in pain processing and a reduction in CS. The exact mechanism of action of tDCS on CS in fibromyalgia is not fully understood. But it is believed to involve the modulation of glutamatergic and GABAergic neurotransmission besides changes in synaptic plasticity.^16^, ^17^

A systematic review and meta‐analysis supported the effectiveness of tDCS in reducing pain intensity in FM.^6^ Factors like the position of electrodes, the number of sessions, stimulation intensity, and associated tasks play a role in the treatment efficacy.^18^ Two montages are used for FM patients, including anodal tDCS over the dorsolateral prefrontal cortex (DLPFC) and M1. Studies demonstrated improving pain and quality of life in both; however, the effects of M1 stimulation are prolonged and can last 30–60 days after the end of treatment.^15^

It has long been established that M1 is responsible for motor control and plays a role in pain modulation. Functional connectivity studies have revealed that M1 is extensively connected with other brain regions, including the somatosensory cortex, thalamus, basal ganglia, cerebellum, and several other cortical areas. These connections are bidirectional, indicating that M1 receives and sends information to different regions. The connectivity of M1 with other parts is critical in the execution of motor tasks and pain modulation.^16^

While single‐session tDCS is generally considered safe and well‐tolerated, there have been several studies have reported adverse effects following tDCS treatment, including headache, skin irritation, tingling sensation, and mood changes. The most common mood changes reported include anxiety, irritability, and emotional lability. However, the onset, duration, and severity are usually mild and transient, with resolution within hours to days after treatment.^13^, ^18^

Previous studies have shown that tDCS can modulate the activity of brain regions associated with mood regulation, including the prefrontal cortex, which has been implicated in the pathophysiology of depression and anxiety. However, the precise mechanisms underlying tDCS‐induced mood changes in fibromyalgia patients are not fully understood. TDCS may have differential effects on mood depending on the patient's baseline mood state or other factors, such as the duration and severity of fibromyalgia symptoms. For instance, patients with a history of bipolar or major depressive disorders showed significant mood changes following the application of tDCS; however, the anodal electrode was placed over DLPFC in those patients, and the adverse effects were temporary.^19^, ^20^ Recent studies have shown that the left frontal cortex (LFC) is involved in emotions and impulse control processing. However, research by Hortensius et al. has found that when the LFC is activated through tDCS, it can lead to greater aggression during outrage episodes. Whereas tDCS has shown promise in the treatment of depression, anxiety, and other disorders, this new study suggests that it may also have unintended consequences for emotional regulation and impulse control.^21^ In general, there is some evidence to suggest that M1‐tDCS can have indirect effects on mood through its impact on neural circuity involved in emotional processing.^22^

In our case, the patient experienced long‐term mood deterioration that lasted for several weeks after one session of tDCS treatment. The exact mechanism of action behind the mood deterioration in our patient is unclear, although there are some possibilities. First, the differences in brain structure or function may make some individuals more susceptible. Second, chronic pain can alter the brain's physiology and increase nervous system sensitivity. Finally, chronic pain patients may have coexisting mental health conditions like depression that can increase the risk of mood changes following tDCS.

In general, transient adverse effects are commonly reported, and if these persist, patients usually ask for help. In this case, he did not revisit us after experiencing those symptoms because he was concerned about going through another treatment and encountering more reactions. Thus, it is necessary to describe the side effects to patients before applying tDCS and carefully monitor patients during and after treatment to identify any adverse effects and provide the timely intervention required.

Further research is needed to understand the potential risks of tDCS for fibromyalgia and identify populations who may be at increased risk for adverse effects.

## AUTHOR CONTRIBUTIONS

**Sarvenaz Rahimibarghani:** Resources; writing – original draft; writing – review and editing. **Hamid R. Fateh:** Conceptualization; supervision; writing – review and editing.

## FUNDING INFORMATION

This work has not received any funding.

## CONFLICT OF INTEREST STATEMENT

The authors have no conflict of interest.

## ETHICS STATEMENT

This study was approved by the Tehran University of Medical Sciences ethical committee.

## CONSENT

Written informed consent was obtained from the patient for publishing this article.

## Data Availability

Data sharing is not applicable to this article as no new data were created or analyzed in this study

## References

[1] Jahan F , Nanji K , Qidwai W , Qasim R . Fibromyalgia syndrome: an overview of pathophysiology, diagnosis and management. Oman Med J. 2012;27(3):192‐195. doi:10.5001/omj.2012.44 22811766 PMC3394355

[2] Thiagarajah AS , Guymer EK , Leech MT , Littlejohn GO . The relationship between fibromyalgia, stress and depression. Int J Clin Rheumatol. 2014;9(4):371‐384. doi:10.2217/ijr.14.30

[3] Nijs J , Lahousse A , Kapreli E , et al. Nociplastic pain criteria or recognition of central sensitization? Pain phenotyping in the past, present and future. J Clin Med. 2021;10(15):3203. doi:10.3390/jcm10153203 34361986 PMC8347369

[4] Rehm S , Sachau J , Hellriegel J , et al. Pain matters for central sensitization: sensory and psychological parameters in patients with fibromyalgia syndrome. Pain Rep. 2021;6(1):e901. doi:10.1097/PR9.0000000000000901 33718743 PMC7952123

[5] Lefaucheur JP , Wendling F . Mechanisms of action of tDCS: a brief and practical overview. Neurophysiol Clin. 2019;49(4):269‐275. doi:10.1016/j.neucli.2019.07.013 31350060

[6] Leite J , Carvalho S , Battistella LR , Caumo W , Fregni F . Editorial: the role of primary motor cortex as a marker and modulator of pain control and emotional‐affective processing. Front Hum Neurosci. 2017;23(11):270. doi:10.3389/fnhum.2017.00270 PMC5440504 28588468

[7] Polanía R , Nitsche MA , Paulus W . Modulating functional connectivity patterns and topological functional organization of the human brain with transcranial direct current stimulation. Hum Brain Mapp. 2011;32(8):1236‐1249. doi:10.1002/hbm.21104 20607750 PMC6870160

[8] Gupta A , Adnan M . Hypomania risk in noninvasive brain stimulation. Cureus. 2018;10(2):e2204. doi:10.7759/cureus.2204 29682434 PMC5908714

[9] Wolfe F , Clauw DJ , Fitzcharles MA , et al. 2016 revisions to the 2010/2011 fibromyalgia diagnostic criteria. Semin Arthritis Rheum. 2016;46(3):319‐329. doi:10.1016/j.semarthrit.2016.08.012 27916278

[10] Jensen KB , Petzke F , Carville S , et al. Anxiety and depressive symptoms in fibromyalgia are related to poor perception of health but not to pain sensitivity or cerebral processing of pain. Arthritis Rheum. 2010;62(11):3488‐3495. doi:10.1002/art.27649 20617526

[11] Yepez D , Grandes XA , Talanki Manjunatha R , Habib S , Sangaraju SL . Fibromyalgia and depression: a literature review of their shared aspects. Cureus. 2022;14(5):e24909. doi:10.7759/cureus.24909 35698706 PMC9187156

[12] Martínez‐Lavín M . Centralized nociplastic pain causing fibromyalgia: an emperor with no cloths? Clin Rheumatol. 2022;41(12):3915‐3917. doi:10.1007/s10067-022-06407-5 36239845 PMC9561334

[13] Brunoni AR , Amadera J , Berbel B , Volz MS , Rizzerio BG , Fregni F . A systematic review on reporting and assessment of adverse effects associated with transcranial direct current stimulation. Int J Neuropsychopharmacol. 2011;14(8):1133‐1145. doi:10.1017/S1461145710001690 21320389

[14] Hortensius R , Schutter DJ , Harmon‐Jones E . When anger leads to aggression: induction of relative left frontal cortical activity with transcranial direct current stimulation increases the anger‐aggression relationship. Soc Cogn Affect Neurosci. 2012;7(3):342‐347. doi:10.1093/scan/nsr012 21421731 PMC3304483

[15] Lloyd DM , Wittkopf PG , Arendsen LJ , Jones AKP . Is transcranial direct current stimulation (tDCS) effective for the treatment of pain in fibromyalgia? A systematic review and meta‐analysis. J Pain. 2020;21(11–12):1085‐1100. doi:10.1016/j.jpain.2020.01.003 31982685

[16] Cagnie B , Coppieters I , Denecker S , Six J , Danneels L , Meeus M . Central sensitization in fibromyalgia? A systematic review on structural and functional brain MRI. Semin Arthritis Rheum. 2014;44(1):68‐75. doi:10.1016/j.semarthrit.2014.01.001 24508406

[17] O'Connell NE , Marston L , Spencer S , DeSouza LH , Wand BM . Non‐invasive brain stimulation techniques for chronic pain. Cochrane Database Syst Rev. 2018;4(4):CD008208. doi:10.1002/14651858.CD008208.pub5 29652088 PMC6494527

[18] Matsumoto H , Ugawa Y . Adverse events of tDCS and tACS: a review. Clin Neurophysiol Pract. 2016;21(2):19‐25. doi:10.1016/j.cnp.2016.12.003 PMC6123849 30214966

[19] Castillo Saavedra L , Mendonca M , Fregni F . Role of the primary motor cortex in the maintenance and treatment of pain in fibromyalgia. Med Hypotheses. 2014;83(3):332‐336. doi:10.1016/j.mehy.2014.06.007 24992875

[20] Utz KS , Dimova V , Oppenländer K , Kerkhoff G . Electrified minds: transcranial direct current stimulation (tDCS) and galvanic vestibular stimulation (GVS) as methods of non‐invasive brain stimulation in neuropsychology—a review of current data and future implications. Neuropsychologia. 2010;48(10):2789‐2810. doi:10.1016/j.neuropsychologia.2010.06.002 20542047

[21] Valle A , Roizenblatt S , Botte S , et al. Efficacy of anodal transcranial direct current stimulation (tDCS) for the treatment of fibromyalgia: results of a randomized, sham‐controlled longitudinal clinical trial. J Pain Manag. 2009;2(3):353‐361. 21170277 PMC3002117

[22] Kuo MF , Nitsche MA . tDCS‐Pharmacotherapy Interactions. In: Brunoni AR , Nitsche MA , Loo CK , eds. Transcranial direct current stimulation in neuropsychiatric disorders. Clinical Principles and Management; 2021:729‐740.

